# Novel Approach Identifies SNPs in *SLC2A10* and *KCNK9* with Evidence for Parent-of-Origin Effect on Body Mass Index

**DOI:** 10.1371/journal.pgen.1004508

**Published:** 2014-07-31

**Authors:** Clive J. Hoggart, Giulia Venturini, Massimo Mangino, Felicia Gomez, Giulia Ascari, Jing Hua Zhao, Alexander Teumer, Thomas W. Winkler, Natalia Tšernikova, Jian'an Luan, Evelin Mihailov, Georg B. Ehret, Weihua Zhang, David Lamparter, Tõnu Esko, Aurelien Macé, Sina Rüeger, Pierre-Yves Bochud, Matteo Barcella, Yves Dauvilliers, Beben Benyamin, David M. Evans, Caroline Hayward, Mary F. Lopez, Lude Franke, Alessia Russo, Iris M. Heid, Erika Salvi, Sailaja Vendantam, Dan E. Arking, Eric Boerwinkle, John C. Chambers, Giovanni Fiorito, Harald Grallert, Simonetta Guarrera, Georg Homuth, Jennifer E. Huffman, David Porteous, Generation Scotland Consortium, The LifeLines Cohort study, The GIANT Consortium, Darius Moradpour, Alex Iranzo, Johannes Hebebrand, John P. Kemp, Gert J. Lammers, Vincent Aubert, Markus H. Heim, Nicholas G. Martin, Grant W. Montgomery, Rosa Peraita-Adrados, Joan Santamaria, Francesco Negro, Carsten O. Schmidt, Robert A. Scott, Tim D. Spector, Konstantin Strauch, Henry Völzke, Nicholas J. Wareham, Wei Yuan, Jordana T. Bell, Aravinda Chakravarti, Jaspal S. Kooner, Annette Peters, Giuseppe Matullo, Henri Wallaschofski, John B. Whitfield, Fred Paccaud, Peter Vollenweider, Sven Bergmann, Jacques S. Beckmann, Mehdi Tafti, Nicholas D. Hastie, Daniele Cusi, Murielle Bochud, Timothy M. Frayling, Andres Metspalu, Marjo-Riitta Jarvelin, André Scherag, George Davey Smith, Ingrid B. Borecki, Valentin Rousson, Joel N. Hirschhorn, Carlo Rivolta, Ruth J. F. Loos, Zoltán Kutalik

**Affiliations:** 1 Department of Genomics of Common Disease, Imperial College London, London, United Kingdom; 2 Department of Medical Genetics, University of Lausanne, Lausanne, Switzerland; 3 Department of Twin Research & Genetic Epidemiology, King's College London, London, United Kingdom; 4 Department of Genetics, Division of Statistical Genomics, Washington University School of Medicine in St. Louis, St. Louis, Missouri, United States of America; 5 MRC-Epidemiology Unit, University of Cambridge, Cambridge, United Kingdom; 6 Interfaculty Institute for Genetics and Functional Genomics, University Medicine Greifswald, Greifswald, Germany; 7 Department of Genetic Epidemiology, University of Regensburg, Regensburg, Germany; 8 Estonian Genome Center, University of Tartu, Tartu, Estonia; 9 Institute of Molecular and Cell Biology, University of Tartu, Tartu, Estonia; 10 Estonian Biocentre, Tartu, Estonia; 11 McKusick-Nathans Institute of Genetic Medicine, Johns Hopkins University School of Medicine, Center for Complex Disease Genomics, Baltimore, Maryland, United States of America; 12 Cardiology, Geneva University Hospitals, Geneva, Switzerland; 13 Epidemiology and Biostatistics, School of Public Health, Imperial College London, London, United Kingdom; 14 Swiss Institute of Bioinformatics, Lausanne, Switzerland; 15 Center for Basic and Translational Obesity Research and Divisions of Endocrinology and Genetics, Boston Children's Hospital, Boston, Massachusetts, United States of America; 16 Metabolism Initiative and Program in Medical and Population Genetics, Broad Institute, Cambridge, Massachusetts, United States of America; 17 Department of Genetics, Harvard Medical School, Boston, Massachusetts, United States of America; 18 Infectious Diseases Service, Department of Medicine, Centre Hospitalier Universitaire Vaudois (CHUV), Lausanne, Switzerland; 19 Department of Health Sciences, University of Milan, Milan, Italy; 20 INSERM-U1061, Montpellier, France; 21 National Reference Network for Orphan Diseases (Narcolepsy and Idiopathic Hypersomnia), Department of Neurology, Gui-de-Chauliac Hospital, Montpellier, France; 22 Queensland Brain Institute, University of Queensland, Brisbane, Australia; 23 Genetic Epidemiology, QIMR Berghofer Institute of Medical Research, Brisbane, Australia; 24 MRC Integrative Epidemiology Unit, University of Bristol, Bristol, United Kingdom; 25 School of Social and Community Medicine, University of Bristol, Bristol, United Kingdom; 26 Diamantina Institute, Translational Research Institute, University of Queensland, Brisbane, Australia; 27 MRC Institute of Genetics and Molecular Medicine, University of Edinburgh, Edinburgh, Scotland, United Kingdom; 28 Department of Medicine, Harvard Medical School, Boston, Massachusetts, United States of America; 29 Department of Genetics, University Medical Center Groningen, University of Groningen, Groningen, The Netherlands; 30 Genomic Variation in Human Populations and Complex Diseases Unit, Human Genetics Foundation and Dept. of Medical Sciences, University of Turin, Turin, Italy; 31 Human Genetics Center, Houston, Texas, United States of America; 32 Research Unit Molecular Epidemiology, Helmholtz Zentrum München, Neuherberg, Germany; 33 Genomic Variation in Human Populations and Complex Diseases Unit, Human Genetics Foundation, Turin, Italy; 34 Centre for Molecular Medicine, Institute of Genetics and Molecular Medicine, University of Edinburgh, Edinburgh, Scotland, United Kingdom; 35 Department of Medicine, Service of Gastroenterology and Hepatology, Lausanne, Switzerland; 36 Neurology Service, Hospital Clinic, Barcelona, Spain; 37 Department of Child and Adolescent Psychiatry, University of Duisburg-Essen, Essen, Germany; 38 Department of Neurology, Leiden University Medical Centre, Leiden, The Netherlands; 39 Sleep Wake Center SEIN, Heemstede, The Netherlands; 40 Division of Immunology and Allergy, Centre Hospitalier Universitaire Vaudois (CHUV), Lausanne, Switzerland; 41 Department of Gastroenterology, University Hospital Basel, Basel, Switzerland; 42 Molecular Epidemiology, QIMR Berghofer Institute of Medical Research, Brisbane, Australia; 43 Sleep and Epilepsy Unit - Clinical Neurophysiology Department, Gregorio Marañón University Hospital, Madrid, Spain; 44 Service of Gastroenterology and Hepatology, Service of Clinical Pathology, Geneva, Switzerland; 45 Institute for Community Medicine, University Medicine Greifswald, Greifswald, Germany; 46 Institute of Genetic Epidemiology, Helmholtz Zentrum München - German Research Center for Environmental Health, Neuherberg, Germany; 47 Institute of Medical Informatics, Biometry and Epidemiology, Chair of Genetic Epidemiology, Ludwig-Maximilians-Universität, Munich, Germany; 48 Cardiovascular Science, National Heart & Lung Institute, Imperial College London, London, United Kingdom; 49 Institute of Epidemiology II, Helmholtz Zentrum München, Neuherberg, Germany; 50 Deutsches Zentrum für Diabetes, Neuherberg, Germany; 51 Institute of Clinical Chemistry and Laboratory Medicine, University Medicine Greifswald, Greifswald, Germany; 52 Institute of Social and Preventive Medicine (IUMSP), Centre Hospitalier Universitaire Vaudois (CHUV), Lausanne, Switzerland; 53 Department of Internal Medicine, Centre Hospitalier Universitaire Vaudois (CHUV), Lausanne, Switzerland; 54 Center for Integrative Genomics (CIG), University of Lausanne, Lausanne, Switzerland; 55 Center for Investigation and Research in Sleep (CIRS), Centre Hospitalier Universitaire Vaudois (CHUV), Lausanne, Switzerland; 56 University of Exeter Medical School, University of Exeter, Exeter, United Kingdom; 57 Institute of Health Sciences, University of Oulu, Oulu, Finland; 58 Biocenter Oulu, University of Oulu, Oulu, Finland; 59 Department of Children and Young People and Families, National Institute for Health and Welfare, Oulu, Finland; 60 Department of Epidemiology and Biostatistics, School of Public Health, MRC-HPA Centre for Environment and Health, Faculty of Medicine, Imperial College London, London, United Kingdom; 61 Unit of Primary Care, Oulu University Hospital, Oulu, Finland; 62 Institute for Medical Informatics, Biometry and Epidemiology, University Hospital of Essen, University of Duisburg-Essen, Essen, Germany; 63 Clinical Epidemiology, Integrated Research and Treatment Center, Center for Sepsis Control and Care (CSCC), Jena University Hospital, Jena, Germany; 64 The Charles Bronfman Institute of Personalized Medicine, The Icahn School of Medicine at Mount Sinai, New York, New York, United States of America; 65 The Mindich Child Health and Development Institute, The Icahn School of Medicine at Mount Sinai, New York, New York, United States of America; 66 The Genetics of Obesity and Related Metabolic Traits Program, The Icahn School of Medicine at Mount Sinai, New York, New York, United States of America; The University of Queensland, Australia

## Abstract

The phenotypic effect of some single nucleotide polymorphisms (SNPs) depends on their parental origin. We present a novel approach to detect parent-of-origin effects (POEs) in genome-wide genotype data of unrelated individuals. The method exploits increased phenotypic variance in the heterozygous genotype group relative to the homozygous groups. We applied the method to >56,000 unrelated individuals to search for POEs influencing body mass index (BMI). Six lead SNPs were carried forward for replication in five family-based studies (of ∼4,000 trios). Two SNPs replicated: the paternal rs2471083-C allele (located near the imprinted *KCNK9* gene) and the paternal rs3091869-T allele (located near the *SLC2A10* gene) increased BMI equally (beta = 0.11 (SD), P<0.0027) compared to the respective maternal alleles. Real-time PCR experiments of lymphoblastoid cell lines from the CEPH families showed that expression of both genes was dependent on parental origin of the SNPs alleles (P<0.01). Our scheme opens new opportunities to exploit GWAS data of unrelated individuals to identify POEs and demonstrates that they play an important role in adult obesity.

## Author Summary

Large genetic association studies have revealed many genetic factors influencing common traits, such as body mass index (BMI). These studies assume that the effect of genetic variants is the same regardless of whether they are inherited from the mother or the father. In our study, we have developed a new approach that allows us to investigate variants whose impact depends on their parental origin (parent-of-origin effects), in unrelated samples when the parental origin cannot be inferred. This is feasible because at genetic markers at which such effects occur there is increased variability of the trait among individuals who inherited different genetic codes from their mother and their father compared to individuals who inherited the same genetic code from both parents. We applied this methodology to discover genetic markers with parent-of-origin effects (POEs) on BMI. This resulted in six candidate markers showing strong POE association. We then attempted to replicate the POE effects of these markers in family studies (where one can infer the parental origin of the inherited variants). Two of our candidates showed significant association in the family studies, the paternal and maternal effects of these markers were in the opposite direction.

## Introduction

The effect of genetic variants on phenotypes may depend upon the parent from whom the variant was inherited [1], [2]. Parent-of-origin effects (POEs) may arise through imprinting; mechanisms of which include cytosine methylation and histone deacetylation [2]. To date around 50 human genes are known to be imprinted and for most mammalian species less than 1% of the genome is confirmed to be imprinted [3]. One plausible explanation for this phenomenon is the parental conflict hypothesis, whereby both parents would like to maximize the influence of their genome on their offspring [4]. Current methods for detecting parent-of-origin effects rely on assigning parental ancestry to the inherited alleles. This is straightforward in linkage studies, which have identified potential POEs on type 2 diabetes, body mass index (BMI) [5], [6], and alcohol intake [7]–[9]. However, only a very few of these findings have been replicated and the identified linkage peaks often span large chromosomal regions harbouring hundreds of genes, hence the causal gene or regulatory sequence is unknown. A notable exception is the work of Kong *et al* [1] who inferred parental origin through genealogy information and long-range phasing to subsequently test for POEs. This study identified six SNPs, four associated with risk of type 2 diabetes and the other two associated with each of breast cancer and basal-cell carcinoma.

Genome-wide association studies (GWASs) of unrelated individuals have very precisely identified a large number of genetic loci harbouring SNPs whose (alternative) allele counts associate with common traits. Since GWASs predominantly include unrelated individuals, the parental origin of the alleles cannot be determined, hence genetic effects influenced by the parental origin of the alleles are typically not considered. Here we present a novel approach that is able to detect POEs using genome-wide genotype data of unrelated individuals. We chose BMI as our target trait, due to previous findings [5], [6] and the large available sample size. We report the discovery of two novel loci affecting BMI in a manner dependent on the parent-of-origin of the transmitted alleles.

## Results

**Figure 1:**
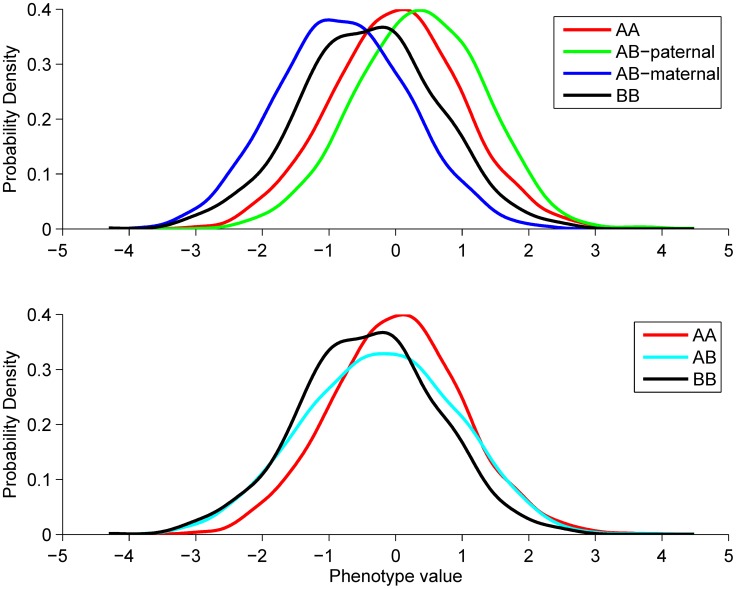
Explanation of the POE test. Top panel illustrates the phenotype distributions in the four genotype groups that would be observed if the parent-of-origin of the alleles were known. Bottom panel shows how these distributions change if the parent-of-origin is unobserved. The resulting heterozygous group will have increased variance due to its heterogeneity. This example describes a scenario we observe for the two replicated hits, namely that the paternal- and maternal effects are of the same size, but opposite in direction (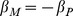). Therefore the average phenotype in the B/B group is the same as in the A/A group, as the paternal and maternal B allele effects cancel each other out. In the A/B group there are two subpopulations: the A-pat/B-mat group with phenotypic mean of 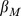 and the A-mat/B-pat group with 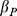 mean. Thus, the two subpopulations combined also have zero mean, but increased variance.

We applied our POE test, which compares the phenotypic variance of the heterozygous genotype group to the variance observed in the homozygous groups, to all SNPs genome-wide. The test, which is applicable to unrelated individuals, assumes that an increased variance in the heterozygous group arises because the heterozygous group consists of two subgroups (paternal reference allele/maternal alternative allele and maternal reference allele/paternal alternative allele) each with different means (see Figure 1). Differences in phenotypic variance were tested using the Brown-Forsythe test, modified to test the mean absolute deviations from the median in the heterozygous and homozygous groups (see Materials and Methods for details). We applied this test to BMI values (corrected for age and age-squared), separately in men and women, in 15 studies, totalling up to 56,092 individuals (detailed description of the cohorts can be found in Tables S1–S3), 13 of which participated in previous meta-analyses of the GIANT consortium [10]. In total 2,673,768 HapMap imputed and genotyped SNPs were tested. For each locus, a lead SNP (with the strongest POE association) was identified; other markers within 1 Mb or in LD (r^2^>0.1) were excluded from further investigations. Sex-specific association summary statistics were then meta-analysed. No sex specific difference in effects were observed, therefore all reported results are sex-combined.

Our criteria to select SNPs to take forward to the replication stage resulted in the selection of six independent SNPs: four lead SNPs with POE P-value <5×10^−6^ and three SNPs in imprinted regions with P<5×10^−4^ (see Fig S1 for QQ-plot), one SNP fulfilled both criteria. See Table 1 for details of these results and Materials and Methods for details of the applied selection methods. These six SNPs were carried forward to the replication stage.

**Table 1:** Discovery POE association results for the six SNPs selected for replication.

SNP	Chr	position (B36)	Gene (distance in kb)	MAF	N (AA+BB)	N (AB)	P-value (POE)	P-value (main)
**Genome-wide (P<5e-6)**								
rs155570	2	59778667	AC007131.1(419)	0.2	32643	15232	2.12E-06	1.70E-03
***rs2471083***	***8***	***140588522***	***KCNK9(105)***	***0.27***	***33885***	***22132***	***9.34E-07***	***8.60E-01***
rs1345919	15	31961131	AVEN(0)	0.22	36850	19142	4.90E-06	5.15E-01
***rs3091869***	***20***	***44859324***	***SLC2A10(61)***	***0.44***	***23891***	***23143***	***4.70E-06***	***3.93E-02***
**Imprinted regions (P<5e-4)**								
rs1398940	4	90156206	FAM13A(0)	0.39	25173	22737	4.50E-04	5.86E-01
rs2471083	8	140588522	KCNK9(105)	0.27	33885	22132	9.34E-07	8.60E-01
rs6500550	16	3686241	TRAP1(0)	0.31	27302	20188	1.69E-04	8.44E-03

SNP matching any of the following two selection criteria were retained: (1) All lead SNPs with POE P-value below 5×10^−6^. (2) All lead SNPs falling in previously reported imprinted regions with POE P-value <5×10^−4^. Note that one SNP is listed twice as it met both criteria. Chr: chromosome, Gene: nearest gene, MAF: minor allele frequency, N (AA+BB): homozygous sample size, N (AB): heterozygous sample size, P-value (main): main effect association P-value in Speliotes *et al*. [10]. The parent of origin P-value, “P-value (POE)”, is the Brown-Forsythe test P-value. SNPs marked in bold are those which replicated in the family studies, see Table 2.

### Replication in family-based studies

**Figure 2:**
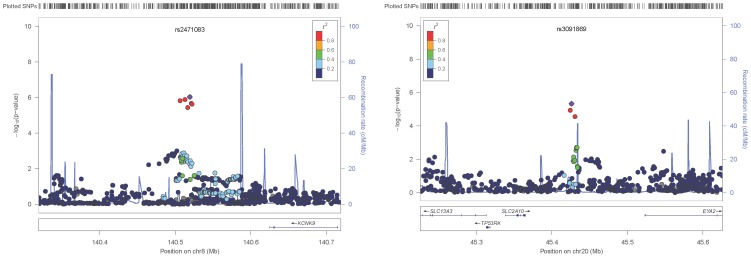
Local association plots. Panels show the local POE association P-values for the *KCNK9* (left panel) and *SLC2A10* (right panel) loci.

The replication stage utilised five family-based studies (see Tables S1–S3) to test for parent-of-origin effects at the six selected SNPs. Only heterozygous individuals are informative when testing for parent-of-origin effects; the number of heterozygous individuals for each of the tested SNPs ranged from 1,122 to 4,128 (see Table 2 and Table S4). A simplified parental asymmetry test (PAT, see Materials and Methods) was applied and SNPs successfully replicated if their PAT P-values were below 0.0083 (Bonferroni corrected significance threshold for family-wise error of 0.05 with six tests). Two of these SNPs, rs2471083 [T/C] (GWAS discovery BMI variance (het vs. hom): 1.058 *vs* 0.963, *P_POE_* = 9.34×10^−7^; replication PAT *P* = 0.00264) and rs3091869 [T/C] (GWAS discovery BMI variance (het vs. hom): 1.046 *vs* 0.957, *P_POE_* = 4.7×10^−6^; replication PAT *P* = 0.00245) successfully replicated. In particular, we found that heterozygous individuals who carry the rs2471083-C allele paternally have 0.11 (SD unit) higher BMI on average than those carrying the C-allele maternally (P = 0.00264). Heterozygous carriers of the paternal rs3091869-C allele have 0.11 (SD unit) lower BMI on average than those carrying the maternal C-allele (P = 0.00245). Figure 2 shows the locuszoom plots of the POE association P-values for the two replicated loci (*KCNK9* and *SLC2A10*).

**Table 2:** Replication of the 6 discovery SNPs in trios (or parent-offspring pairs).

SNP	Coded allele	Other allele	beta (coded paternal) - beta (coded maternal)					P-value for beta difference					beta meta	P meta	N meta
			QIMR	FAMHS	FHS	ALSPAC	GS	QIMR	FAMHS	FHS	ALSPAC	GS			
rs155570	G	A	0.218	−0.033	−0.098	0.23	0.136	5.82E-03	7.97E-01	1.72E-01	2.43E-02	2.32E-01	0.079	5.57E-02	1646∶1093
rs1345919	A	T	−0.17	−0.167	0.098	−0.032	−0.006	3.72E-02	2.06E-01	1.56E-01	7.15E-01	9.54E-01	−0.03	4.46E-01	1231∶1696
rs1398940	G	A	0.104	0.033	0.098	−0.07	0.008	1.41E-01	7.57E-01	9.50E-02	2.70E-01	9.30E-01	0.038	2.45E-01	1984∶2144
***rs2471083***	***C***	***T***	***0.012***	***0.064***	***0.24***	***0.007***	***0.135***	***8.81E-01***	***5.62E-01***	***1.48E-04***	***9.31E-01***	***1.89E-01***	***0.112***	***2.64E-03***	***1453∶1959***
***rs3091869***	***C***	***T***	***−0.107***	***−0.152***	***−0.117***	***−0.062***	***−0.199***	***1.19E-01***	***1.29E-01***	***2.75E-01***	***3.35E-01***	***5.05E-02***	***−0.112***	***2.45E-03***	***1477∶1696***
rs6500550	T	C	0.053	−0.306	0.039	−0.169	−0.03	4.64E-01	3.14E-03	5.79E-01	2.22E-02	7.29E-01	−0.057	1.08E-01	1952∶1484

For each target SNP, in each family we searched for trios (or parent-offspring pairs) with heterozygous offspring and determined the parent of origin of the alleles (whenever possible). From each family at most one heterozygous offspring with known parental origin was then collected and grouped according to the parental origin of the alleles. The equality of phenotypic means in the two groups was tested using a Student t-test (“P-value for beta difference column”). The difference between the phenotypic means were meta-analysed using inverse-variance weighting. Table notations: “beta meta” and “P meta”: meta-analysis estimate of beta (coded paternal) - beta (coded maternal) effect size differences and the corresponding P-value, “N meta”: total number of heterozygous offspring with (coded-maternal/other-paternal), (coded-paternal/other-maternal) genotypes, respectively.

### Impact of the discovered variants

By combining the effect difference estimates 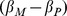 from the family-based studies and the marginal association effect sizes 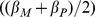 from the largest-to-date meta-analytic study on BMI [10], we estimated the effects of the maternal and paternal alleles. For both rs2471083-C and rs3091869-T we obtained 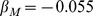 and 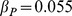. Using these effect sizes and the population frequency of these SNPs, we calculated the explained variance of these SNPs (if their parent of origin is known) to be 0.24% and 0.30% for rs2471083 and rs3091869, respectively. These effects are comparable to that of the strongest BMI-associated variant in the *FTO* gene (0.34%) [10].

Notably, rs2471083 is located 105 kb upstream of the imprinted gene *KCNK9*. Mutations in this potassium channel gene cause Birk-Barel syndrome, a maternally transmitted syndrome of mental retardation, hypotonia, and unique dysmorphism, resulting from genomic-imprinting [11]. SNPs within 2 kb have been shown to be associated with HDL cholesterol, adiponectin levels [12] and blood pressure [13]. Its impact on hypertension is potentially via a mechanism involving aldosterone, the concentration of which correlates strongly with fat mass. Interestingly, *KCNK9* knock-out mice exhibited more fragmented sleep episodes [14] and 7.1%–9.6% increased weight gain (P = 0.02) at 19–20 weeks of age [15]. SNP rs3091869 is 61 kb upstream of *SLC2A10*, a glucose transporter involved in arterial morphogenesis. SNPs in low LD (in CEU *r^2^* = 0.05) with rs3091869 have been shown to alter body fat distribution [16].

We tested these two confirmed SNPs in 705 trios with paediatric (extreme) obese offspring in which parental origin of the alleles was known in up to 255 individuals [17]. No significant effect was observed (see Table S5). This could be due to insufficient power, different genetic mechanisms between young individuals and adults or that our association is specific to variations within the range of normal BMI.

### Expression experiments

We evaluated whether the parent of origin effect of the rs2471083-T and rs3092611-T (proxy for rs3091869-T, r^2^ = 0.998) alleles can be observed in the expression levels of their respective genes (*KCNK9* or *SLC2A10*). To test this we carried out quantitative PCR (qPCR) experiments using lymphoblastoid cell lines (LCL) of the CEPH families. These cell lines have been used extensively to identify imprinted genes [18], [19]. Using the available trio data we could infer the parental origin of the alleles of rs2471083 and rs3092611 in 33 (9 maternal T alleles, 24 paternal T alleles) and 24 (16 maternal T alleles, 8 paternal T alleles) individuals respectively (Table S7). We performed between 2 and 10 technical replicates per individual (mean of 7.75) and samples with high coefficient of variation (>5%) were discarded in order to ensure robustness. After quality control, 124 expression values from 23 (mat:pat = 4∶19) samples for *KCNK9* and 240 expression values from 24 (mat:pat = 16∶8) for *SLC2A10* were available for analysis. We fitted a linear mixed model to test for association between expression levels (Ct values) and allelic origin. The paternal T allele of rs2471083 was associated with lower *KCNK9* expression levels (+1.08 [SD unit] Ct values, P = 0.0096), and the paternal T allele of rs3092611 was associated with higher *SLC2A10* expression values (−1.09 [SD unit] Ct values, P = 0.0023). To ensure there was no systematic bias in our experiments giving rise to spurious POE associations we repeated the qPCR experiments for two housekeeping genes *GAPDH* and *HRPT1*. Both analyses gave non-significant POE P-values (P>0.3).

### Methylation lookups

POEs can be driven by differences between inherited paternal and maternal methylation. To explore whether the observed parent-of-origin effects at our discovered SNPs were driven by differential methylation we tested whether methylation in the regions (Chr8: 140.45–140.65 Mb and Chr20: 45.3–45.55 Mb) was (i) associated with the two respective SNPs (rs2471083, rs3091869) in 262 unrelated individuals from the TwinsUK cohort and (ii) associated with BMI in two independent cohorts: 79 BMI discordant (difference >0.5 SD) monozygotic twin pairs from the TwinsUK cohort and a sample of 412 unrelated individuals from the EPIC-Italy cohort. None of these analyses showed significant association (see Supplemental Data S1, Figures S2, S3 and Table S8 for further details).

## Discussion

Our novel approach revealed two SNPs, located near the genes *KCNK9* and *SLC2A10*, influencing BMI in a parent-of-origin specific fashion. These loci were the first and fourth most significant genome-wide in our new POE test for unrelated individuals and both showed significant parent-of-origin effects in family studies. Both SNPs exhibit polar overdominance, where homozygous individuals have equal (baseline) phenotypes and heterozygous genotypes confer relative risk/protection, depending on the parental origin. Polar overdominance, has been observed in humans for type2 diabetes [1] and BMI [20], however it is very rare and its molecular mechanism is unknown.

RT-PCR experiments revealed that gene expression levels of *KCNK9* and *SLC2A10* in LCLs were also influenced in a parent-of-origin manner. The expression of these genes is highest in the brain (although it is also expressed in testis, liver, colon, adrenal gland and kidney; see http://www.genecards.org/) indicating a potential neuronal involvement. Expression levels of *KCNK9* and *SLC2A10* in living brain cells might have been more informative and robust, however, such information is not available. The applied qPCR method was optimised to ensure that the expression levels measured in LCLs were representative only of the target transcript and amplification efficiency was assessed to be sensitive enough to allow the detection of even small changes in gene expression. Interestingly, rs2471083 alleles, regardless of their parental origin, show marginally significant association (P = 0.03) with *KCNK9* expression levels in the hippocampus (http://www.broadinstitute.org/gtex/). Our methylation analyses did not reveal any evidence that the POEs were driven by differences in inherited paternal and maternal methylation. Neither of our two SNPs tag common copy number variants (CNVs) (based on the CNV reference data used in Heid *et al*. [21]) and we found only one sample (out of 14,315 available in-house, whose BMI Z-score was +1.18) with a 76 kb deletion overlapping rs2471083. Hence, the effect of the two discovered SNPs are unlikely to be driven by CNVs. To check whether the two confirmed SNPs (rs3091869, rs2471083), or SNPs in LD (10 with *r^2^*>0.8 in 1000 Genomes EUR population), show regulatory activity, we queried RegulomeDB (http://regulome.stanford.edu). None of these SNPs were annotated to have more than minimal binding evidence (RegulomeDB score below 4).

A previous study proposed to detect POE in inbred F_2_ mice by a two-component mixture distribution fitting of the heterozygous genotype group and further two components for the homozygous groups [22]. This method requires a parametric distribution of the phenotype to be assumed, small violations of this assumption can result in heavily biased parameter estimates. The method we chose is more robust to a wide range of phenotype distributions (due to the underlying Brown-Forsythe test employed), computationally faster (making it attractive for testing millions of SNPs) and applicable to probabilistic genotype calls. Our POE test for unrelated GWAS samples is similar to a test proposed to detect gene-environment interactions [23] in that it exploits differences in phenotypic variance to detect a phenomenon not directly measured. Inflated phenotypic variance in the heterozygous group might also be the result of other phenomenon: (i) a phenotype altering effect (be it genetic or environmental) acting only on the heterozygous group; (ii) an overdominant effect combined with a genetic or environmental interaction or non-linear, monotonic phenotype transformation that has different derivatives for low and high trait values; (iii) a large marginal additive effect combined with a (monotonic) transformation for which the second derivative is maximised at the mean phenotype value of the heterozygous group (see Materials and Methods for details). More generally, the combination of the scale on which the phenotype is measured and a strong marginal association with an allelic dosage may give rise to spurious associations using variance tests [24]. Recently some evidence has emerged about loci which effect the variance of phenotypes (through impacting environmental plasticity, canalization, developmental stability, etc.) that can be detected via association with phenotypic variability [25]. Therefore, the top hits obtained by our POE test may need further prioritisation before proceeding to trio-based confirmation. We recommend the following checks: (a) Exclude SNPs with overdominant effects; (b) For SNPs with low POE P-value, test gene-environment (GxE) interaction (as done in [23]) via modelling phenotypic variance as a function of the genotype dosage (coded in additive, recessive or dominant fashion). If this test is more significant than the POE test, it is probably a GxE that is driving the POE association and also as a side effect we will observe significant difference in the variance between the two homozygous groups. (c) If a SNP with low POE P-value has marginal effect on the trait, repeat the POE test for various transformed versions of the phenotype such as log and inverse-normal quantile. If the resulting POE P-values are not robust, give lower priority to the examined SNP.

For our confirmed SNPs multiple lines of evidence show that the parent-of-origin effects are real, most convincingly clear replication in independent family data of parent-of-origin associations of the hit SNPs with both BMI and gene expression levels. Further, the GWAS discovery associations are very unlikely to be artefacts of the factors discussed above: (i) there is no evidence of overdominant, additive, recessive or dominant effects (the mean BMI values are near identical in the three genotype groups), hence the signals cannot be driven by gene-environment interactions or be an artefact of the scale on which the phenotype is measured (ii) no SNP within 500 kb has any detectable marginal effect on BMI thus the association cannot be driven by haplotype-specific marginal effects [26]; (iii) the phenotypic variances in the two homozygous groups, are almost identical (rs2471083: 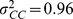, 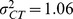, 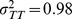 and rs3091869: 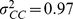, 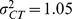, 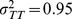); (iv) POE test with log- and inverse-normal quantile transformed BMI values resulted in similar results (Table S6), further reducing the likelihood of an artefact resulting from the scale on which the phenotype is measured [24].

Some of the negative results of the other SNPs carried forward to the replication phase in the family data could be explained by lack of power. The power to replicate POE associations in family-based studies is dependent on the available number of heterozygous individuals (for details see Supplemental Data S1) and thus increases with minor allele frequency (MAF). Therefore, it is unsurprising that the two SNPs which replicated had relatively high MAF (>27%).

Linkage studies have identified four regions exerting POE on BMI (10p12, 12q24, 13q32) [5] and 2q31 [27]). We looked up SNPs in these regions in our genome-wide discovery POE association results. The reported linkage regions showed enrichment for lower than expected POE P-values (see Figure S4 for regional QQ-plots), however, no SNPs survived Bonferroni correction. We also tried to replicate a SNP in exon 5 of *DLK1* (rs1802710) because this SNP showed polar overdominance for obesity in children [20], but only a very slight trend (*P* = 0.32) was visible in our study. Previously reported BMI-associated loci [10] show some enrichment for lower POE P-values (Supplemental Data S1, Tables S9, S10 and Figure S5), however these need to be replicated in family studies.

Previous work comparing strength of associations of mother-offspring BMI with father-offspring BMI did not reveal intrauterine influence on obesity in children [28]. A similar conclusion was reached in a systematic review of seven studies [29], while stronger maternal influence was observed in a recent longitudinal study [30]. The difference in conclusions may be due to that fact that the former studies included predominantly older children than the longitudinal study (0–3.5 years). At early age the diet of the offspring may be more similar to that of the mother than the father (e.g. due to breastfeeding), which might have contributed to the higher mother-offspring BMI similarity found by Linabery *et al.* [30].

In summary, our findings indicate that POEs may play a role in adult obesity. The two identified SNPs have strong parent-of-origin effect on BMI, close to that of the *FTO*, contributing substantially to the heritability of BMI. Our follow-up experiments demonstrated parent-of-origin specific gene expression modulation, but failed to link methylation activity of these loci to BMI values. Inevitably for newly discovered loci, further studies are warranted to determine how these variations functionally influence obesity in humans. The reliance of our approach on difference in phenotypic variance means that it cannot be extended to binary outcomes. Since there are other phenomena which can give rise to significant POE association, we recommend that top hits from our method are followed up in family studies, where parental origin of alleles can be inferred. In addition, our variance based POE test for GWAS data is naturally much less powerful than actually testing the mean values of the two heterozygous subgroups in trios. However, GWASs of unrelated individuals are several-fold more numerous and typically much larger than studies with a trio design, hence our methodology provides a great advance in parent-of-origin research by providing means to exploit all available GWAS data of unrelated individuals in order to identify parent-of-origin effects on continuous phenotypes.

## Materials and Methods

### Ethics statement

All participating studies were approved by the respective institutional Ethics Committees. All study participants gave written consent including for genetic studies.

### Detecting parent of origin effects

If we denote the alleles of a bi-allelic SNP by “A” (reference) and “B” (alternative) the possible genotypes are A/A, A/B and B/B. Standard GWASs estimate the effect of the alternative allele dosage on the phenotype in question. In this work we are interested in associations in which a phenotype (*y*) is influenced by the alleles of a particular SNP and the effect depends on the parental origin of these alleles. In the presence of a parent-of-origin effect the heterozygous genotype group is split into two subgroups, depending on the parental origin of the A and B alleles. We assume that the phenotype of any individual in the A/A genotype group is modelled by 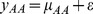, where 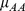
is the mean and 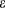
is an individual level error with mean zero and variance 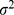. If the maternal and paternal effects of the B allele are 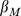
and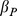, it follows that the phenotype of an individual in the B/B group is 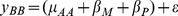 and its variance is 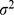. (Note that as a consequence the maternal and paternal effects of the A allele are 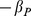 and 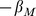.) Here we assume 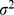
is constant across genotype groups (A/A, A/B and B/B) and 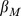
and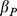 are fixed effects. The effects of violations of these assumptions are covered in the discussion. The phenotype in the heterozygous group is a 50%–50% mixture of two distributions (Fig 1a): 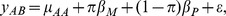
where 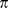 is a Bernoulli random variable (with parameter ½), taking values 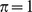 if the B allele is inherited from the mother and 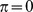 if inherited from the father. The heterozygous phenotype distribution can be simplified to 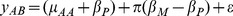 Since 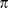 and 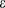 are independent random variables, the phenotypic variance of the heterozygous genotype group 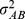 is
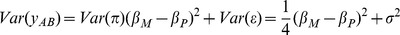



If a parent-of-origin effect is present 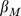
and
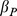
are different, thus 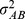 is larger than the variance observed in the homozygous groups (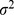) (Figure 1). Therefore, although in regular GWAS data we cannot identify the two subgroups within A/B genotypes, we can detect POE via increased phenotypic variance in the heterozygous group relative to the homozygous groups.

In the presence of a marginal association a phenotype transformation could alter the genotype group variances and introduce bias into the test [24]. For this reason we analysed untransformed age-, age^2^-corrected BMI values (normalised to have zero mean and unit variance) separately for men and women. Standard variance tests (such as the F-test) are, however, sensitive to deviations from the Gaussian distribution.

Therefore, we used a robust version of the Brown-Forsythe test. Briefly, we first centred the phenotype values (at zero) in each genotype group to avoid inflated variance in the presence of marginal effects in the group of all homozygote individuals. We denote these centred phenotypes by 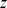, where 
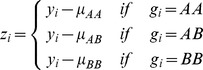



Here 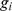 stands for the genotype of individual 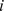, and 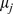 represents the median phenotype value in genotype group 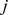, where 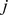 can take the values of AA, AB or BB. We then regress the absolute deviations from the median onto a 0–1 coded genotype group identifier (1 for heterozygous and 0 for homozygous individuals) in order to estimate the POE effect size [31]. This regression result in a slope estimate
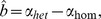
where 
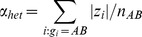
 and 
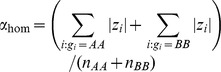
. The corresponding standard error is
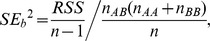
where
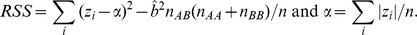



Finally, the POE P-value is assigned based on the test statistic 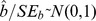. The test was extended to imputed genotype probabilities and implemented in the latest version (v0.98) of the Quicktest software (http://www3.unil.ch/wpmu/sgg/quicktest/). The robustness of this test to deviations from normality has been studied in [32] and its power in [31].

### SNP selection strategy

We applied our POE test genome-wide to all HapMap imputed markers in a set of cohorts and results were combined across cohorts using fixed-effect inverse-variance weighting meta-analysis. SNPs were selected for replication if they met at least one of the following two criteria: (1) POE P-value <5×10^−6^ or (2) POE P-value <5×10^−4^ and within 500 kb of previously reported imprinted regions according to the Catalogue of Parent of Origin Effects database (http://igc.otago.ac.nz/home.html). At loci which met either criteria, a lead SNP (with the strongest POE association) was identified; other markers within 1 Mb or in LD (*r^2^*>0.1) were excluded from further investigations. In total 2,673,768 HapMap imputed and genotyped SNPs were analysed, of which 29,457 were considered as lying in imprinted regions, criterion (2). Using the procedure of Gao *et al*. [33] we estimated the effective number of tests considered by each criterion to be ∼1,000,000 and 6,100 respectively, justifying the ∼100 fold drop in the P-value threshold applied to the second criterion.

### Testing in family-based studies

We tested our findings in family-based studies using a simplified parental asymmetry test [34] (PAT). For each target SNP, in each family we searched for trios (or parent-offspring pairs) with heterozygous offspring and determined the parent of origin of the alleles (whenever possible, i.e. at least one homozygous parent). From each family at most one heterozygous offspring with known parental origin was then collected and grouped according to the parental origin of the alleles. Note that although POE is acting in every genotype group, it can only be detected in the heterozygous group.

As at the discovery phase, we used sex-, age- and age^2^-corrected BMI residuals as phenotype. The equality of phenotypic means in the two groups was tested using a Student t-test. When significant differences were detected we also estimated the difference between paternal and maternal effect sizes, which is simply the difference between the phenotype averages in the paternal- and maternal- groups.

### Effect size estimation

In order to estimate paternal 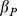) and maternal (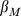) effect sizes it is sufficient to know their mean 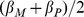 and their difference 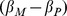. The difference between paternal and maternal effect alleles can also be derived from GWAS of unrelated individuals. It is easy to see that the test statistic defined as
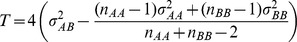
gives an unbiased estimate of 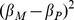. Since 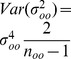, the variance of T is 
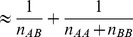
. Therefore, the absolute difference in paternal and maternal effects (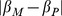) can be estimated if the phenotypic variances in the three genotype groups are known. However, these estimates will be strongly subject to the winner's curse [35], thus we used the family studies to derive more reliable estimates of 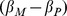. To reduce the effect of differences in the distribution of BMI between the family-based studies, we meta-analysed the difference estimates 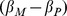 from each study in order to obtain a combined estimate of 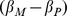. The average of the maternal- and paternal effects, 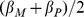, is the association effect size using a simple additive genetic model, which can be most accurately estimated from the largest-to-date meta-analytic study on BMI [10] (including ∼250,000 individuals).

### Effect of phenotype transformation in case of marginal association

If there is an additive marginal genetic effect influencing the trait certain transformations may inflate the phenotypic variance of the heterozygous group. Let 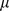 be the phenotypic mean in the heterozygous group and 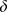 the marginal effect of the SNP (on the original scale). Let 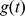 denote an S-shaped transformation function of the form 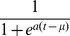 that is applied to the trait.

In the following we show that for any 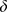 value arbitrarily large phenotypic variance inflation can be achieved in the heterozygous group, compared to the two homozygous groups by an appropriate parameter choice for 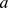. Using a second order Taylor expansion the variance of the transformed phenotype in the heterozygous group can be estimated by
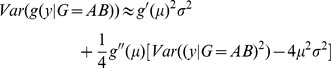



If we assume the phenotype follows a Gaussian distribution 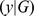 then, 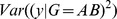 simplifies to 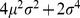 and thus
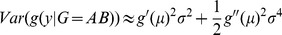



Without loss of generality one can assume that 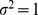. The variance in AA genotype group can be estimated similarly and thus
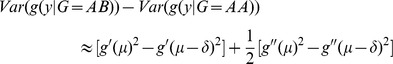



Using the special form of 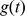, the variance difference can be expressed as
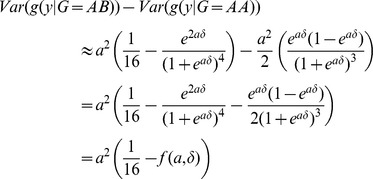
and since 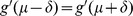 and 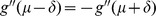


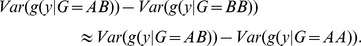



It is easy to see that for a fixed 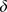


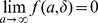



As 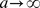, 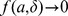 faster than 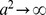, thus for any effect size 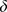 we can find a transformation function 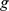 such that the variance inflation of the heterozygous group exceeds any arbitrary threshold.

### Cell lines, nucleic acids isolation, sequencing and qPCR

Lymphoblastoid cell lines were derived from peripheral blood leukocytes of 95 members of 11 CEPH families [36] (#102, #884, #1333, #1340, #1341, #1345, #1346, #1347, #1362, #1408, #13292). They were purchased from the Coriell Cell Repository (http://ccr.coriell.org/), and cultured as previously described [37]. DNA was extracted by using the QIAamp DNA Mini kit (QIAGEN), and RNA by using the RNeasy Mini kit (QIAGEN), according to the manufacturer's instructions. Primer sequences were designed to amplify a 328-bp region on chromosome 8 that spans the rs2471083 polymorphism (forward primer: 5′-ACCACAGAAGTCAGTAGACGAG-3′; reverse primer: 5′- GTGACATTGGGAGCATGGGA-3′) and a 146-bp region on chromosome 20 that spans the rs3092611 polymorphism (forward primer: 5′-GCCACCAGTGGTCTGATAGT-3′; reverse primer: 5′- TAACTCGTCATTCTGCCCTGG -3′). PCR amplification was performed in a 25 µl reaction using GoTaq polymerase (Promega). After purification of PCR products (ExoSAP-IT, USB), sequencing reactions were carried out by using 1 µl of each of the 3.2 µM sequencing primers and 0.5 µl of BigDye Terminator v1.1 (Applied Biosystems). Following on-column purification (EdgeBio), sequencing products were run on an ABI-3130 XLS sequencer (Applied Biosystem). To synthesize cDNA, 2 µg of total RNA was retrotranscribed using the Superscript III reverse transcriptase (Invitrogen/Life Technologies) according to the manufacturer's instructions and a mix of random hexamers and oligo-dT that facilitate the detection of poorly expressed genes. To validate primers for qPCR, we first performed a series of test amplifications by using a defined range of primer concentrations (50–200 nM). We then loaded 10 µl of each qPCR product on 1% agarose gels to check the specificity of the amplification product, which should correspond to a 113-bp (*KCNK9*) and 148-bp (*SLC2A10*) fragment. To test *KCNK9* and *SLC2A10* PCR efficiency a standard curve made of five serial dilutions of brain and lung cDNA were used, respectively, since the two genes are known to be highly expressed in these organs. We obtained a standard curve slope of −3.49 for *KCNK9* and of −3.37 for *SLC2A10*, corresponding to 94% and 98% PCR efficiency. For more details see Supplemental Data S1.

### Comparing Ct values

The output of the analysis was threshold cycles (Ct), i.e. the number of cycles at which the fluorescent signal of the reaction crosses a pre-determined threshold value. Since standard quantification methods (including normalization by housekeeping genes) introduce a considerable amount of experimental noise for very lowly expressed genes, raw Ct values were used to perform an absolute quantification of *KCNK9* and *SLC2A10* transcripts. As negative controls, housekeeping genes (*HPRT1*, *GAPDH*) were also tested for parent-origin-effect to exclude the possibility that the observed difference in *KCNK9* and *SLC2A10* expression levels was due to the sample preparation process. Raw Ct values were inverse-normal quantile transformed and a linear mixed model was fitted (using the R function *lmer*) modelling the technical replicates as random effects and parental origin as a fixed effect.

## Supporting Information

Figure S1 QQ-plot of the POE test P-values for SNPs in imprinted regions (left) and for the whole genome (right).
(PNG)

Figure S2 Left hand side plots describe SNP-methylation associations (mQTLs), where each point is a methylation probe. X-axis represents their physical position and y-axis the −log10 association P-value with the target SNP, whose location is indicated by the dashed line. Note that rs3092611 was used as a proxy for rs3091869 (r^2^ = 0.998). The corresponding QQ-plots appear on the right hand side. Neighbouring methylation probes are strongly correlated therefore expected P-values were computed by estimating the effective number of tests For expected P-values we computed the effective number of tests [33].
(PNG)

Figure S3 Left hand side plots describe methylation associations with BMI in MZ twins. Each point is a methylation probe, X-axis represents their physical position and y-axis the −log10 association P-value with BMI. Location of the target SNP is indicated by the dashed line. Note that rs3092611 was used as a proxy for rs3091869 (r^2^ = 0.998). The corresponding QQ-plots appear on the right hand side. Neighbouring methylation probes are strongly correlated therefore expected P-values were computed by estimating the effective number of tests For expected P-values we computed the effective number of tests [33].
(PNG)

Figure S4 POE association results for previously reported imprinted BMI linkage regions. Second dashed line corresponds to the Benjamini-Hochberg 5% FDR threshold.
(PNG)

Figure S5 POE association P-value QQ-plot for the top 58 independent SNPs with marginal BMI-association P-value <10^−5^ in Speliotes *et al*. [10].
(PNG)

Table S1 Description of the BMI distribution in the participating cohorts.
(XLSX)

Table S2 Brief summary of the participating cohorts.
(XLSX)

Table S3 Information on the genotyping methods in the participating cohorts.
(XLSX)

Table S4 Replication of the 6 discovery SNPs in family studies. Extended version of Table 2.
(XLSX)

Table S5 Parental asymmetry test results for our 6 candidate SNPs in adolescent population.
(XLSX)

Table S6 Analysis of the effect of gender- and age-correction and phenotype transformations on the POE association results.
(XLSX)

Table S7 Genotyping results and parental allele determination for members of the CEPH families.
(XLSX)

Table S8 Association results between methylation- and BMI- differences among MZ twins of the TwinsUK study.
(XLSX)

Table S9 POE association P-values for the 32 SNPs associated with BMI in the largest-to-date meta-analysis [10].
(XLSX)

Table S10 POE association P-values for the top 58 independent SNPs with BMI-association P-value <10^−5^ in the largest-to-date meta-analysis [10].
(XLSX)

Data S1 Supporting information contain text on the expression- and methylation analysis, the effective sample size derivation in mother-offspring vs trio studies, POE look-ups for SNPs with marginal BMI association, study descriptions and acknowledgements.
(DOCX)
