# Supplementary figures and images for: Novel Approach Identifies SNPs in SLC2A10 and KCNK9 with Evidence for Parent-of-Origin Effect on Body Mass Index
Source: PLoS Genet. 2014 Jul 31;10(7):e1004508. doi: 10.1371/journal.pgen.1004508 (PMC4117451; doi:10.1371/journal.pgen.1004508)

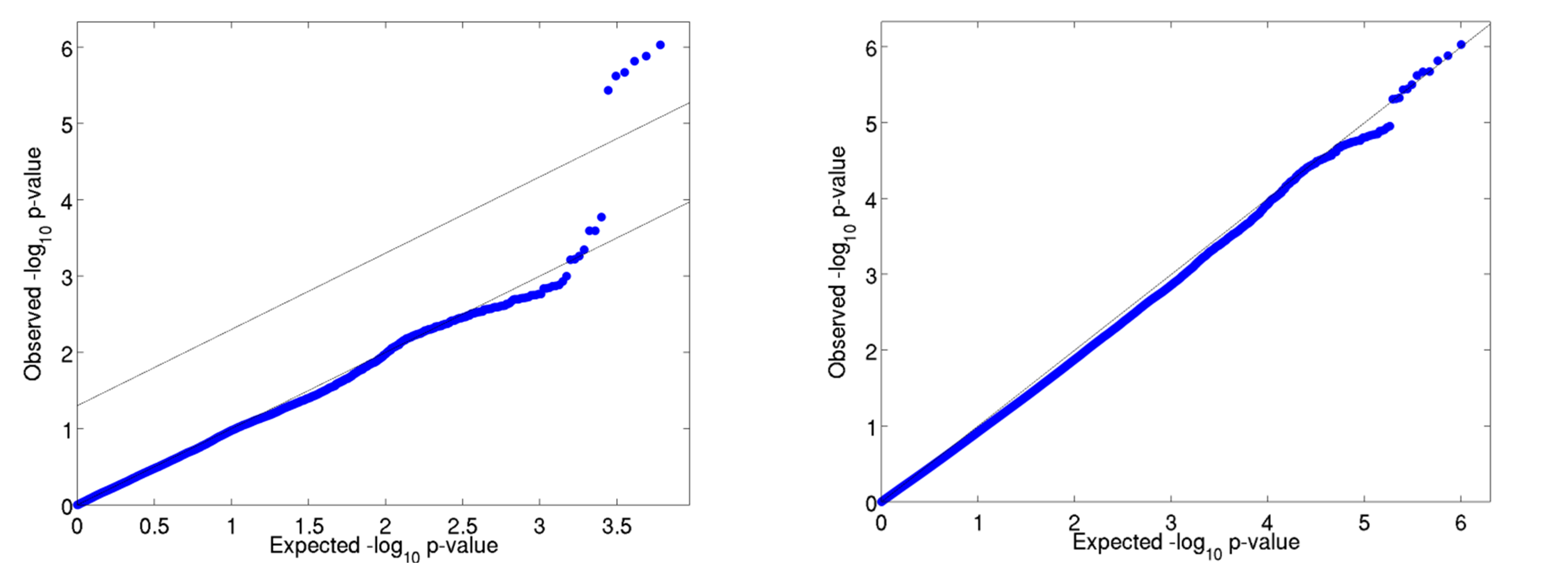

Supplement: Figure S1 — QQ-plot of the POE test P-values for SNPs in imprinted regions (left) and for the whole genome (right). (PNG) [file pgen.1004508.s001.png]

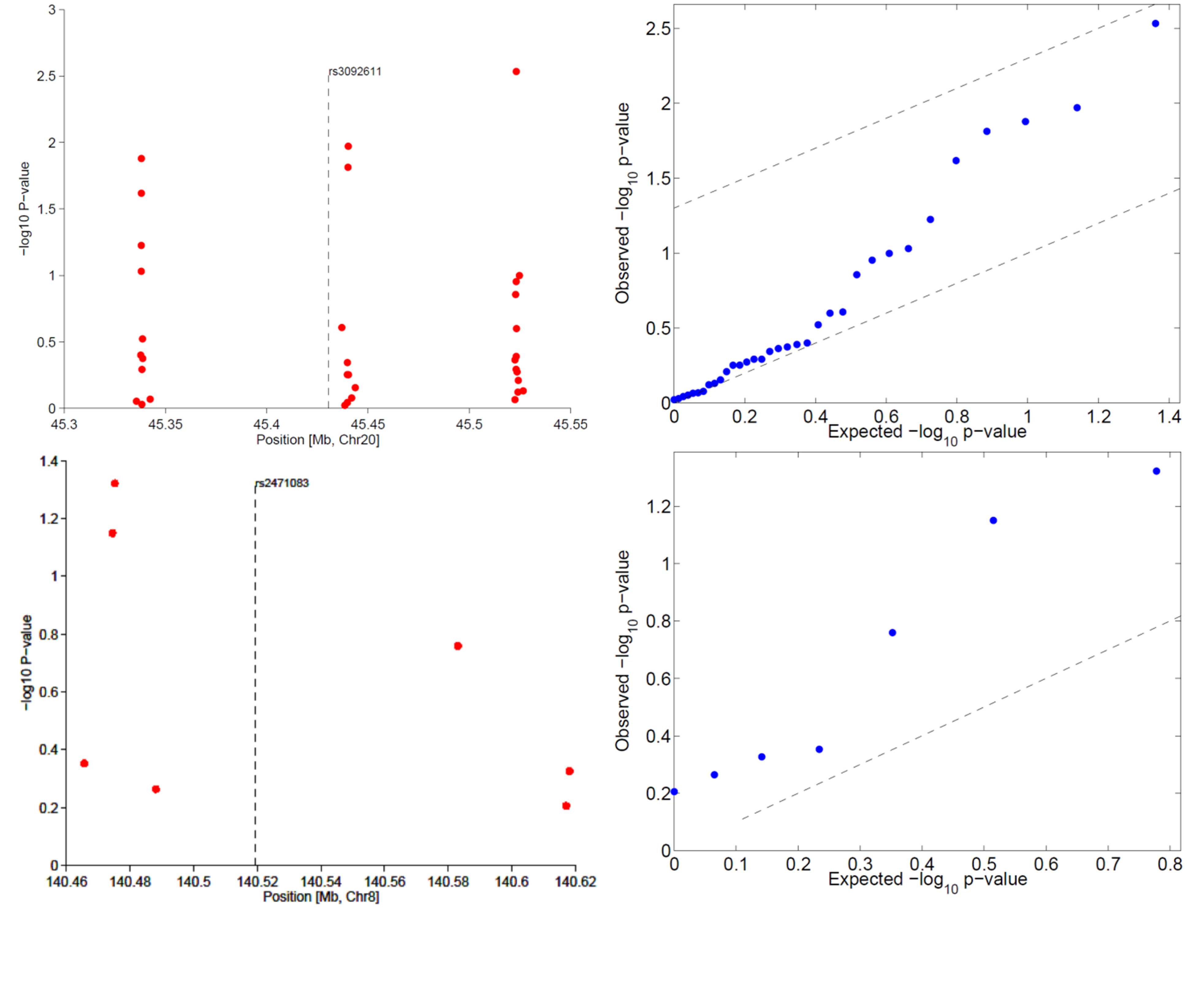

Supplement: Figure S2 — Left hand side plots describe SNP-methylation associations (mQTLs), where each point is a methylation probe. X-axis represents their physical position and y-axis the −log10 association P-value with the target SNP, whose location is indicated by the dashed line. Note that rs3092611 was used as a proxy for rs3091869 (r2 = 0.998). The corresponding QQ-plots appear on the right hand side. Neighbouring methylation probes are strongly correlated therefore expected P-values were computed by estimating the effective number of tests For expected P-values we computed the effective number of tests [33]. (PNG) [file pgen.1004508.s002.png]

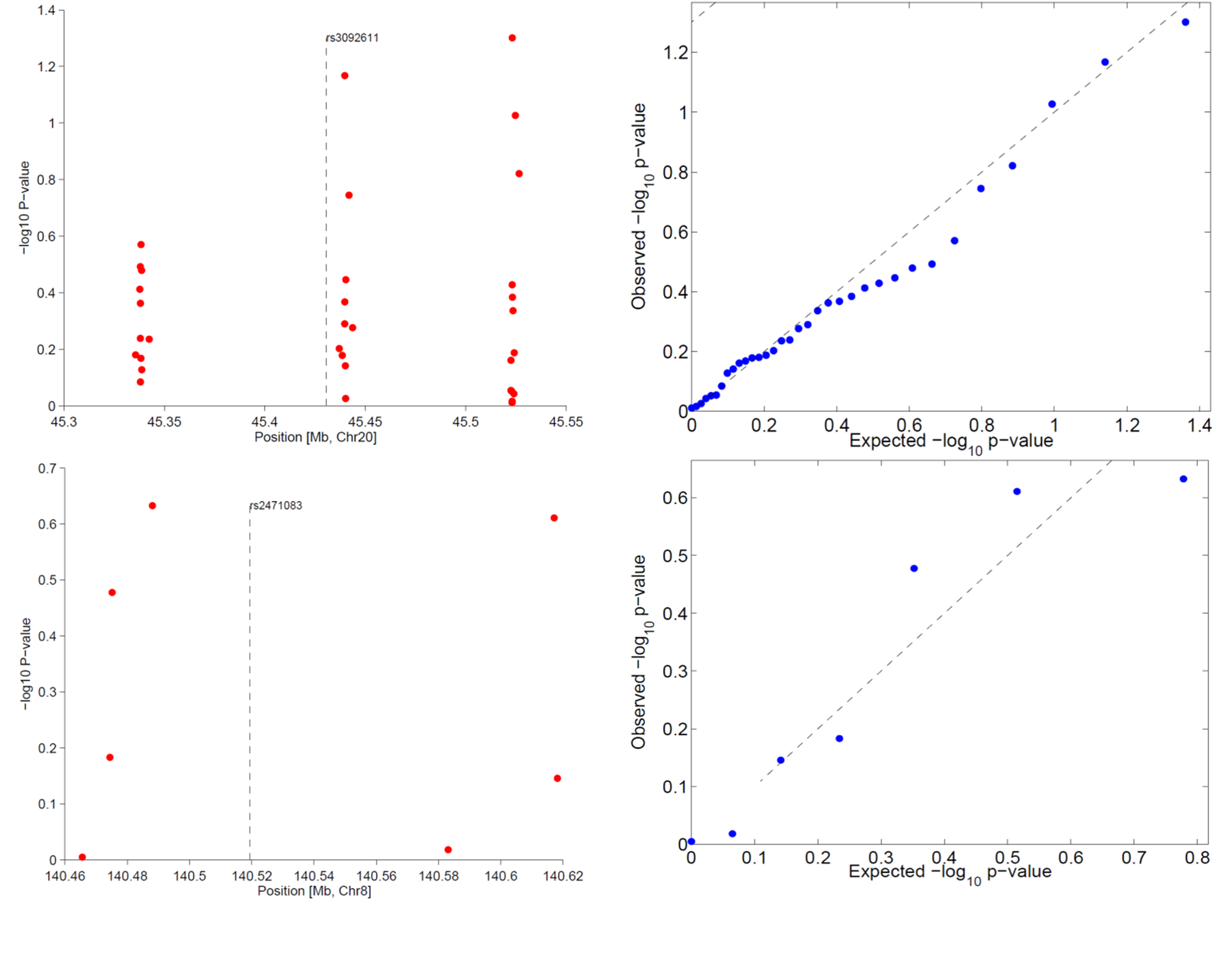

Supplement: Figure S3 — Left hand side plots describe methylation associations with BMI in MZ twins. Each point is a methylation probe, X-axis represents their physical position and y-axis the −log10 association P-value with BMI. Location of the target SNP is indicated by the dashed line. Note that rs3092611 was used as a proxy for rs3091869 (r2 = 0.998). The corresponding QQ-plots appear on the right hand side. Neighbouring methylation probes are strongly correlated therefore expected P-values were computed by estimating the effective number of tests For expected P-values we computed the effective number of tests [33]. (PNG) [file pgen.1004508.s003.png]

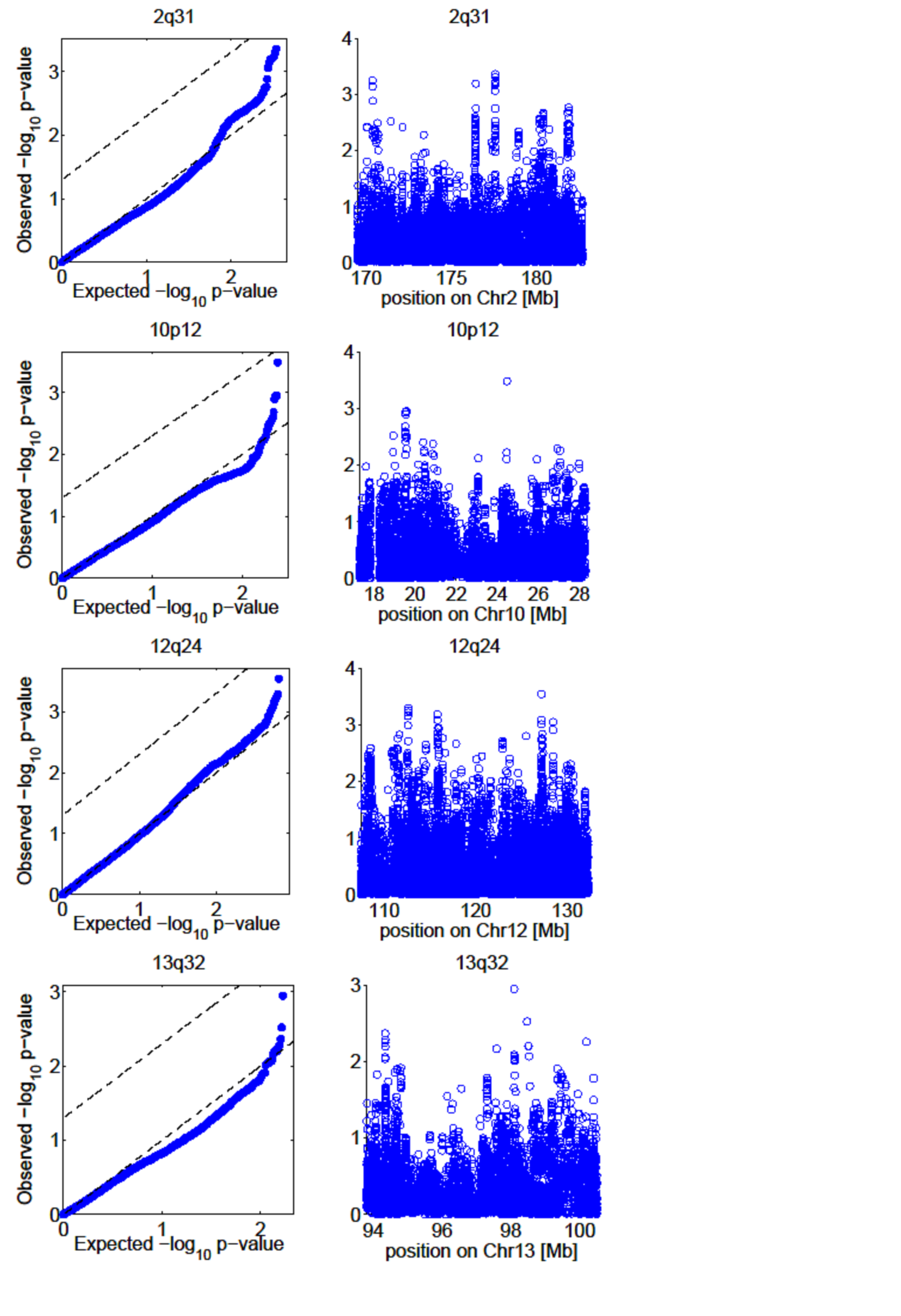

Supplement: Figure S4 — POE association results for previously reported imprinted BMI linkage regions. Second dashed line corresponds to the Benjamini-Hochberg 5% FDR threshold. (PNG) [file pgen.1004508.s004.png]

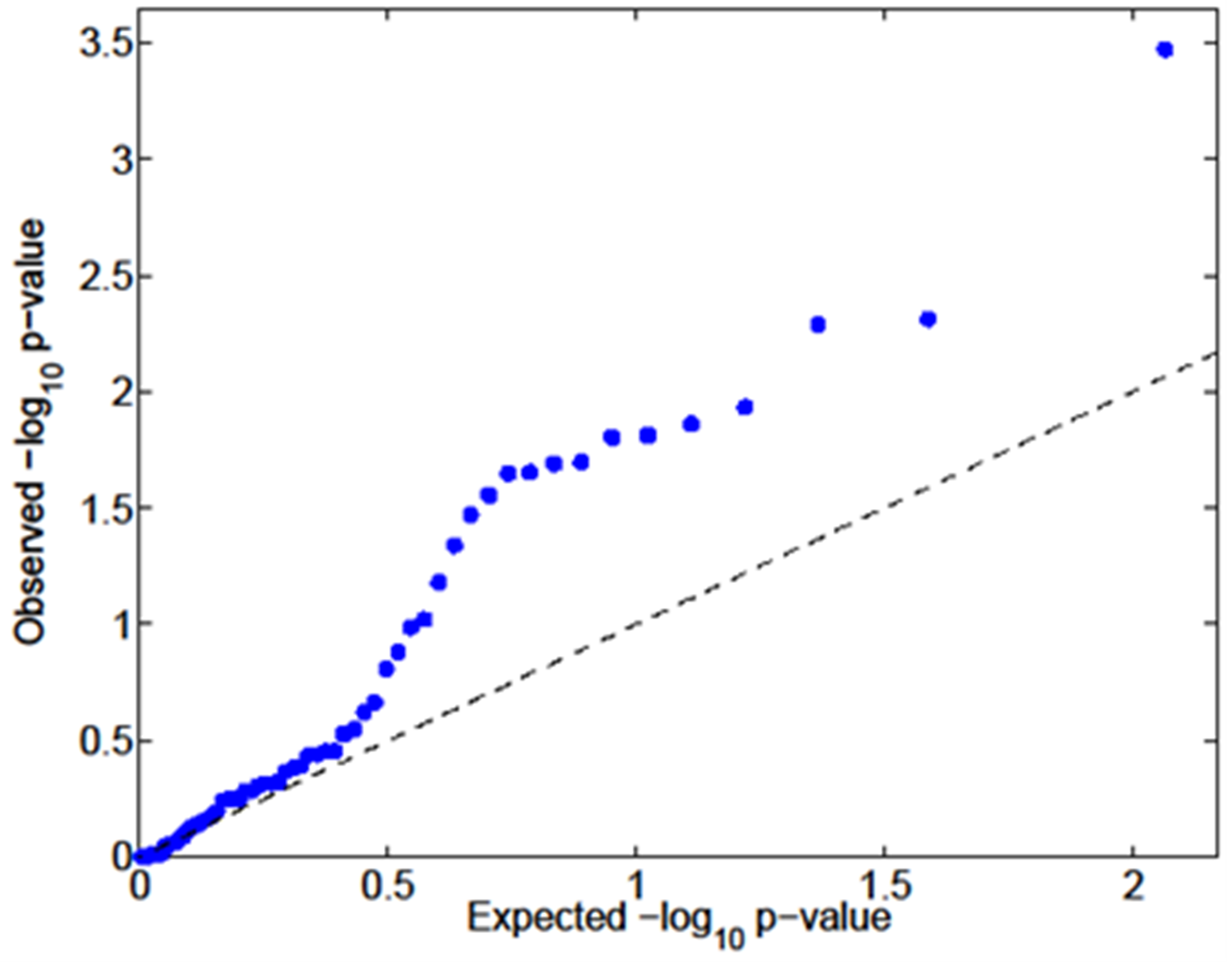

Supplement: Figure S5 — POE association P-value QQ-plot for the top 58 independent SNPs with marginal BMI-association P-value <10−5 in Speliotes et al. [10]. (PNG) [file pgen.1004508.s005.png]
