# Supplementary material for: Novel Approach Identifies SNPs in SLC2A10 and KCNK9 with Evidence for Parent-of-Origin Effect on Body Mass Index
Source: PLoS Genet. 2014 Jul 31;10(7):e1004508. doi: 10.1371/journal.pgen.1004508 (PMC4117451; doi:10.1371/journal.pgen.1004508)
Supplement: Data S1 — Supporting information contain text on the expression- and methylation analysis, the effective sample size derivation in mother-offspring vs trio studies, POE look-ups for SNPs with marginal BMI association, study descriptions and acknowledgements. (DOCX) [file pgen.1004508.s016.docx]

***Supplemental Data***

**Novel method identifies SNPs in *SLC2A10* and *KCNK9* exhibiting parent-of origin effect on body mass index**

Clive J Hoggart, Giulia Venturini, Massimo Mangino, Felicia Gomez, Giulia Ascari, Jing Hua Zhao, Alexander Teumer, Thomas W Winkler, Natalia Tšernikova, Jian'an Luan, Evelin Mihailov, Georg B Ehret, Weihua Zhang, David Lamparter, Tõnu Esko, Aurelien Macé, Sina Rüeger, Pierre-Yves Bochud, Matteo Barcella, Yves Dauvilliers, Beben Benyamin, David M Evans, Caroline Hayward, Mary F Lopez, Lude Franke, Alessia Russo, Iris M Heid, Erika Salvi, Sailaja Vendantam, Dan E Arking, Eric Boerwinkle, John C Chambers, Giovanni Fiorito, Harald Grallert, Simonetta Guarrera, Georg Homuth, Jennifer E Huffman, David Porteous, Generation Scotland Consortium, The LifeLines Cohort study, The GIANT Consortium, Darius Moradpour, Alex Iranzo, Johannes Hebebrand , John P Kemp, Gert J Lammers, Markus H Heim, Nicholas G Martin, Grant W Montgomery, Rosa Peraita-Adrados, Joan Santamaria, Francesco Negro, Carsten O Schmidt, Robert A Scott, Tim D Spector, Konstantin Strauch, Henry Völzke, Nicholas J Wareham, Wei Yuan, Jordana T Bell, Aravinda Chakravarti, Jaspal S Kooner, Annette Peters, Giuseppe Matullo, Henri Wallaschofski, John B Whitfield, Fred Paccaud, Peter Vollenweider, Sven Bergmann, Jacques S Beckmann, Mehdi Tafti, Nicholas D Hastie, Daniele Cusi, Murielle Bochud, Timothy M Frayling, Andres Metspalu, Marjo-Riitta Jarvelin, André Scherag, George Davey Smith, Ingrid B Borecki, Valentin Rousson, Joel N Hirschhorn, Carlo Rivolta, Ruth JF Loos, Zoltán Kutalik

1. **Expression analysis**

***Cell lines and extraction of nucleic acids***

Lymphoblastoid cell lines were derived from peripheral blood leukocytes of 95 members of 11 CEPH families16 (#102, #884, #1333, #1340, #1341, #1345, #1346, #1347, #1362, #1408, #13292). They were purchased from the Coriell Cell Repository (http://ccr.coriell.org/), and cultured as previously described17. Nucleic acids were isolated from 10 million cells. DNA was extracted by using the QIAamp DNA Mini kit (QIAGEN), and RNA by using the RNeasy Mini kit (QIAGEN), according to the manufacturer’s instructions. For total RNA purification the protocol was implemented by an on-column DNAse treatment in order to completely eliminate residual DNA traces. Concentration of nucleic acids was measured with the NanoDrop ND-1000 spectrophotometer.

***DNA amplification and sequencing reactions***

Primer sequences were designed using the Primer-BLAST software to amplify a 328-bp region on chromosome 8 that spans the rs2471083 polymorphism (forward primer: 5’-ACCACAGAAGTCAGTAGACGAG-3’; reverse primer: 5’- GTGACATTGGGAGCATGGGA-3’) and a 146-bp region on chromosome 20 that spans the rs3092611 polymorphism (forward primer: 5’-GCCACCAGTGGTCTGATAGT-3’; reverse primer: 5’- TAACTCGTCATTCTGCCCTGG -3’). PCR amplification was performed in a 25 μl reaction containing 20 ng genomic DNA, 1x GoTaq buffer, 1.2 mM MgCl2, 0.1 mM dNTPs, 0.4 μM of each primer, and 0.01 U/μl of GoTaq polymerase (Promega). Amplification conditions were: an initial step at 95°C for 2 minutes, followed by 35 cycles of denaturation at 94°C for 30 seconds, annealing at 60°C for 30 seconds, and extension at 72°C for 1 minute. Before the end of the reaction, a final extension step at 72°C for 5 minutes was performed. After purification of PCR products (ExoSAP-IT, USB), sequencing reactions were carried out by using 1 μl of each of the 3.2 μM sequencing primers (5’- ATCTCAGATGTGTGTCCTA-3’ and 5’- GTGACATTGGGAGCATGGGA-3’ for the rs2471083 polymorphism and 5’- TAACTCGTCATTCTGCCCTGG -3’ for the rs3092611 polymorphism) and 0.5 μl of BigDye Terminator v1.1 (Applied Biosystems). Following on-column purification (EdgeBio), sequencing products were run on an ABI-3130 XLS sequencer (Applied Biosystem).

***cDNA synthesis and quantification by real-time PCR***

To synthesize cDNA, 2 μg of total RNA was retrotranscribed using the Superscript III reverse transcriptase (Invitrogen/Life Technologies) according to the manufacturer’s instructions and a mix of random hexamers and oligo-dT that facilitate the detection of poorly expressed genes. After cDNA synthesis, an RNase H treatment (Invitrogen/Life Technologies) was performed to remove RNA. The primer sequences used to amplify the KCNK9 gene (forward: 5’- CACGGTCATCACCACCATAG-3’; reverse: 5’- GGAACATGACCAGTGTCAGC-3’) and the SLC2A10 gene (forward: 5’-GGAAAGTTTGTCCGGCG-3’; reverse: 5’-CAGCAAAGACACAGAGGCAC-3’) were from the qPrimerDepot database (http://primerdepot.nci.nih.gov/) and were specifically designed to span exon-exon junctions, thus avoiding genomic DNA to be amplified during the real-time PCR (qPCR) reactions. To validate these primers, we first performed a series of test amplifications by using a defined range of primer concentrations (50-200 nM). We then loaded 10 µl of each qPCR product on 1% agarose gels to check the specificity of the amplified DNA, which should correspond to a 113-bp (KCNK9) and 148-bp (SLC2A10) fragment. A standard curve made of five serial dilutions of cDNA was used to test the efficiency of the primer pairs. To test KCNK9 and SLC2A10 PCR efficiency brain and lung cDNA were used, respectively, since the two genes are known to be highly expressed in these organs. We obtained a standard curve slope of -3.49 for KCNK9 and of -3.37 for SLC2A10, corresponding to 94% and 98% PCR efficiency. KCNK9 and SLC2A10 cDNA were amplified using the Sybr Green PCR Master mix (Applied Biosystem). qPCR reactions were performed using standard amplification conditions, except for the number of PCR cycles, for which we used 60.

Comparative analysis of DNA microarrays, pyrosequencing and quantitative PCR suggested that these techniques yield similar measures of gene expression[[1](#_ENREF_1)]. Since in our lab has extensive expertise with qPCR, we chose this latter technique.

1. **Methylation analysis**

We focused on methylation levels in our two POE associated regions: Chr8: 140.45-140.65Mb and Chr20: 45.3-45.55Mb. Methylation profiles were assessed in the TwinsUK and the EPIC-Italy cohorts in peripheral blood leukocytes using Infinium HumanMethylation450K BeadChip array (<http://www.illumina.com/products/methylation_450_beadchip_kits.ilmn>).

***mQTLs.*** We first selected unrelated individuals in the TwinsUK cohort to compare and genotype dosage of the two discovered hits. Association between methylation levels in the Chr 8 region and genotype dosage of rs2471083 and methylation levels in the Chr 20 region and rs3092611 were tested in 262 unrelated individuals from the TwinsUK cohort using linear regression. None of them seemed to be clearly associated with methylation patterns in the TwinsUK cohort (Figure S2) or in the HapMap samples[[2](#_ENREF_2)].

***Methylation vs BMI in BMI discrepant MZ twins*.** Association between BMI and methylation was tested in 79 BMI-discordant (>0.5SD BMI difference) monozygotic twins, using linear regression. We observed no link between methylation- and BMI differences in these MZ twin pairs (Figure S3).

***Methylation vs BMI regardless of genetic background.*** Methylation activity in the EPIC-Italy cohort, measured on the same platform, in the two regions of interest was tested for association with BMI in 412 unrelated individuals. We found no evidence linking BMI with methylation of these regions.

1. **Effective sample size in mother-offspring vs trio studies**

We could not fully exploit the ALSPAC study because only mother-offspring pairs were genotyped, instead of complete trios. The power of the family-based test to detect POE is a monotone increasing function of the test statistic, which is proportional to $\sqrt{Nf_{AB}f_{BA}}$, where *N* is the total number of offspring and *f_AB_* are the proportion of those for whom it can be inferred they inherited the A allele from the mother and B from the father (A-mat/B-pat), similarly and vice versa for *f_BA_*. We denote the frequency of the A allele by *p* and that of the B allele by *q = 1-p*.

Maternal A, paternal B can only be inferred if the mother is homozygous AA, thus

*f_AB_ = Pr(A-mat/B-pat | mother is AA) * Pr(mother is AA)=q p^2^*

Similarly *f_BA_=p q^2^*. Therefore the test statistic is proportional to $\sqrt{N(pq)^{3}}$.

With complete trios A-mat/B-pat can be inferred if either parent is homozygous. If we denote this event by E and its complement, both parents being heterozygous by F, then

*f_AB_ = Pr(A-mat/B-pat, E) = Pr(A-mat/B-pat) – Pr(A-mat/B-pat, F )*

*= Pr(A-mat/B-pat) – Pr(A-mat/B-pat | F ) * Pr(F )= pq – (1/4)*(2pq)^2^= pq(1-pq).*

Similarly *f_BA_ = pq(1-pq)*, therefore the test statistic is proportional to $\sqrt{N(pq)^{2}(1-pq)^{2}}$.

Thus, for the allele frequency range of our selected top hit SNPs (*q* = 0.2-0.44), a 1.52-2.1 fold drop could be observed in the test statistic for the same number of offspring when we use mother-offspring pairs instead of trios.

1. **Look-up in BMI-associated loci**

We also tested whether any of the previously identified BMI-associated GWAS SNPs[[3](#_ENREF_3)] show significant POE. No individual SNP survived Bonferroni correction (see Table S9), but we did find significant enrichment of low P-values among these SNPs: seven out of the 32 have nominally significant (*P_POE_* < 0.05) parent-of-origin effect (Binomial test P = 1.39x10^-4^). Since intermediate significant BMI-associated loci are enriched for true effects, we repeated the exercise for the top 58 lead SNPs with P<10^-5^ in the Speliotes *et al.*[[3](#_ENREF_3)] paper. At this threshold the enrichment became more striking (14/58 had nominally significant POE, binomial P = 1.16x10^-7^), and one SNP (rs4883723) 400kb upstream of the *OLFM4* gene survived Bonferroni correction (Table S9). Further, rs4883723 is in very high LD (*r^2^* = 0.92) with a SNP reported to be associated with extreme childhood obesity[[4](#_ENREF_4)]. These results indicate that many known BMI-associated loci may exhibit POEs. Family genotype data will be required to confirm if any of these SNPS do indeed exhibit parent of origin effects.

1. **New study descriptions**

**ALSPAC**

The study website contains details of all the data that is available through a fully searchable data dictionary http://www.bris.ac.uk/alspac/researchers/data-access/data-dictionary.

**EPICOR-Turin**

We performed an epigenome-wide analysis on DNAs extracted from 412 blood samples using the [Infinium HumanMethylation450 BeadChip](http://www.illumina.com/products/methylation_450_beadchip_kits.ilmn) by Illumina. All individuals belong to the EPICOR study from the Italian cohort of the European Prospective Investigation into Cancer and Nutrition (EPIC) project. All subjects were healthy at recruitment; 206 individuals developed myocardial infarction (MI) during follow-up and 206 subjects were healthy matched controls. BMI and methylation data were available for all the 412 participants. The aim of this study was to assess the interaction among diet, lifestyle, biomarkers and molecular risk factors in the etiology of CVD.

1. **Study acknowledgements**

**ALSPAC:** We are extremely grateful to all the families who took part in this study, the midwives for their help in recruiting them and the whole ALSPAC team, which includes interviewers, computer and laboratory technicians, clerical workers, research scientists, volunteers, managers, receptionists and nurses. This publication is the work of the authors, and they will serve as guarantors for the contents of this paper.

**CoLaus:** The authors also express their gratitude to the participants in the Lausanne CoLaus study and to the investigators who have contributed to the recruitment, in particular the co PIs, Peter Vollenweider, Vincent Mooser, and research nurses of CoLaus: Yolande Barreau, Anne-Lise Bastian, Binasa Ramic, Martine Moranville, Martine Baumer, Marcy Sagette, Jeanne Ecoffey, Sylvie Mermoud.

**EPIC:** We acknowledge the contribution of the staff and participants of the EPIC-Norfolk Study.

**FamHS:** We would like to thank Avril Adelman for her technical assistance with this project.

**Fenland:** We are grateful to all the volunteers for their time and help, and to the General Practitioners and practice staff for assistance with recruitment. We thank the Fenland Study Investigators, Fenland Study Co-ordination team and the Epidemiology Field, Data and Laboratory teams.

**HYPERGENES:** The HYPERGENES consortium members who took part included: 1) University of Milano and Fondazione Filarete with Daniele Cusi, Project Coordinator, Cristina Barlassina, Erika Salvi, Sara Lupoli, Maurizio Marconi, Gianna Petrini, Vincenzo Toschi; 2) Katholieke Universiteit Leuven, with Jan Staessen, Jan Staessen, Tatiana Kuznetsova, Lutgarde Thijs; 3) Jagiellonian University Medical College, Krakow, with Kalina Kawecka-Jaszcz, Katarzyna Stolarz, Agnieszka Olszanecka, Wiktoria Wojciechowska; 4) IBM Israel – Science and Technology LTD, with Amnon Shabo, Ariel Frakash, Simona Cohen, Boaz Carmeli, Dan Pelleg, Michal Rosen-Zvi, Hani Neuvrith-Telem; 5) I.M.S. – Istituto di Management Sanitario S.r.l., Milan, with Pietro Conti, Costanza Conti, Mariella D’Alessio; 6) Institute of Internal Medicine, Siberian Branch of Russian Academy of Medical Sciences, Novosibirsk, with Yuri Nikitin, Sofia Malyutina, M. Voevoda, Andrew Ryabikov, E. Pello, Maxim Ryabikov; 7) Imperial College of Science, Technology and Medicine, with Paolo Vineis and Clive J Hoggart; 8) INSERM – Institut National de la Santé et de la Recherche Médicale U772, with Xavier Jeunemaitre, Pierre-François Plouin, Anne-Paule Gimenez-Roqueplo, Rosa Vargas-Poussou, Geneviève Beaurain; 9) University of Warwick. Cardiovascular Medicine & Epidemiology Group, Clinical Sciences Research Institute, with Francesco P Cappuccio, Michelle A Miller, Chen Ji; 10) Università degli Studi di Sassari. Hypertension and cardiovascular prevention centre, with Nicola Glorioso, Chiara Maria Troffa, Giuseppe Argiolas, Francesca Fau, Silvia Pitzoi; 11) STMICROELECTRONICS SRL, with Enrico Rosario Alessi; 12) Universite de Lausanne. Department of Medical Genetics, with Carlo Rivolta, Jacques S. Beckmann, Zoltan Kutalik, Paola Benaglio; 13) Pharnext S.A.S., Paris, with Daniel Cohen and Ilya Chumakov; 14) Softeco Sismat Spa, Genova, with Stefano Bianchi; 15) Shanghai Institute of Hypertension, with Jiguang Wang and Li Yan; 16) Charles University in Prague. Department of Internal Medicine II, Pilsen, with Jan Filipovsky, Otto Mayer, Milan Hromadka, Jitka Seidlerova, Milena Dolejsova, Lukas Handl; 17) Università degli Studi di Padova. Department of Clinical and Experimental Medicine, with Edoardo Casiglia, Valerie Tikhonoff, Laura Schiavon, Anna Bascelli, Elisa Pagnin; 18) Medical University of Gdansk. Hypertension Unit, Department of Hypertension and Diabetology, with Krzysztof Narkiewicz, Marzena Chrostowska, Radoslaw Szczech, Michal Hoffmann; 19) University Vita-Salute San Raffaele, with Paolo Manunta, Chiara Lanzani, Maria Teresa Sciarrone, Lorena Citterio, Laura Zagato. Carlo Rivolta was supported by the Swiss National Science Foundation (Grant 310030_138346) and the Gebert Rüf Foundation, Switzerland (Rare Diseases - New Technologies grant).

**LifeLines:** We thank Behrooz Alizadeh, Annemieke Boesjes, Marcel Bruinenberg, Noortje Festen, Pim van der Harst, Ilja Nolte, Lude Franke, Mitra Valimohammadi for their help in creating the GWAS database, and Rob Bieringa, Joost Keers, René Oostergo, Rosalie Visser, Judith Vonk for their work related to data-collection and validation. The authors are grateful to the study participants, the staff from the LifeLines Cohort Study and Medical Biobank Northern Netherlands, and the participating general practitioners and pharmacists. The LifeLines Cohort study comprises of the following collaborators: Alizadeh B, de Boer R, Boezen HM, Bruinenberg M, Franke L, van der Harst P, Hillege H, van der Klauw M, Navis G, Ormel J, Postma D, Rosmalen J, Slaets J, Snieder H, Stolk R, Wolffenbuttel B, Wijmenga C.

**LOLIPOP**: We thank the participants and research staff who made the study possible.

**NFBC66:** The authors would like to acknowledge the contribution of the late Academian of Science Leena Peltonen.

**QIMR**: We are grateful to the twins and their families for their generous participation in these studies. We would like to thank staff at the Queensland Institute of Medical Research: Dixie Statham, Ann Eldridge and Marlene Grace for sample collection, Grant Montgomery, Anjali Henders, Lisa Bowdler, Steven Crooks and staff of the Molecular Epidemiology Laboratory for sample processing and preparation, Scott Gordon for data QC and preparation, and David Smyth and Harry Beeby for IT support.

**TwinsUK:** The study was funded by the Wellcome Trust; European Community’s Seventh Framework Programme (FP7/2007-2013). The study also receives support from the National Institute for Health Research (NIHR)- funded BioResource, Clinical Research Facility and Biomedical Research Centre based at Guy's and St Thomas' NHS Foundation Trust in partnership with King's College London. SNP Genotyping was performed by The Wellcome Trust Sanger Institute and National Eye Institute via NIH/CIDR.

**References**

1. Wittkopp PJ, Haerum BK, Clark AG (2006) Parent-of-origin effects on mRNA expression in Drosophila melanogaster not caused by genomic imprinting. Genetics 173: 1817-1821.

2. Bell JT, Pai AA, Pickrell JK, Gaffney DJ, Pique-Regi R, et al. (2011) DNA methylation patterns associate with genetic and gene expression variation in HapMap cell lines. Genome Biol 12: R10.

3. Speliotes EK, Willer CJ, Berndt SI, Monda KL, Thorleifsson G, et al. (2010) Association analyses of 249,796 individuals reveal 18 new loci associated with body mass index. Nat Genet 42: 937-948.

4. Bradfield JP, Taal HR, Timpson NJ, Scherag A, Lecoeur C, et al. (2012) A genome-wide association meta-analysis identifies new childhood obesity loci. Nat Genet 44: 526-531.
